# 

**Published:** 1907-01-05

**Authors:** 


### BOOKS RECEIVED.

THE F. A. DAVIS CO., PHILADELPHIA.

"Materia Medica and Therapeutics." 6th edition. By Dr. Shoemaker.

BAILLIÈRE, TINDALL AND COX.

"Aids to Medical Diagnosis." By A. J. Whiting, M.D.

CASSELL AND CO., LTD.

"Tumours, Innocent and Malignant." 4th edition. By J. Bland Sutton, F.R.C.S.

### NOTICE TO CORRESPONDENTS.

All MSS., Letters, Books for Review, and other matters intended for the Editor should be addressed THE EDITOR, "THE HOSPITAL," THE HOSPITAL BUILDINGS, 28 & 29 SOUTHAMPTON STREET, STRAND, W.C.

The Editor cannot undertake to return rejected MS., even when accompanied by stamped directed envelope.

### Notice to Advertisers and Subscribers.

All Advertisements, Orders for Copies of THE HOSPITAL, and Business Communications should be addressed to **THE MANAGER (Not to the Editor),** THE HOSPITAL, 28 & 29 Southampton Street, Strand London, W.C.

The Cost of Subscription, post free, is as follows:—Per week, 3½d.; per quarter, 3s. 3d.; per half-year, 6s. 6d.; per annum, 13s. Foreign and Colonial:—Per annum, 19s. 6d., payable, in all cases, in advance.

